# An Impedance-Loaded Surface Acoustic Wave Corrosion Sensor for Infrastructure Monitoring

**DOI:** 10.3390/s24030789

**Published:** 2024-01-25

**Authors:** Jagannath Devkota, David W. Greve, Nathan Diemler, Richard Pingree, Ruishu Wright

**Affiliations:** 1 National Energy Technology Laboratory, 626 Cochran Mill Road, Pittsburgh, PA 15236, USArichard.pingree@netl.doe.gov (R.P.); ruishu.wright@netl.doe.gov (R.W.); 2 NETL Support Contractor, 626 Cochran Mill Road, Pittsburgh, PA 15236, USA; 3 Presently with the National Institute for Occupational Safety and Health, Pittsburgh, PA 15236, USA; 4 Department of Electrical and Computer Engineering, Carnegie Mellon University, 5000 Forbes Avenue, Pittsburgh, PA 15213, USA

**Keywords:** corrosion, sensor, surface acoustic wave, delay line, pipeline

## Abstract

Passive surface acoustic wave (SAW) devices are attractive candidates for continuous wireless monitoring of corrosion in large infrastructures. However, acoustic loss in the aqueous medium and limited read range usually create challenges in their widespread use for monitoring large systems such as oil and gas (O&G) pipelines, aircraft, and processing plants. This paper presents the investigation of impedance-loaded reflective delay line (IL-RDL) SAW devices for monitoring metal corrosion under O&G pipeline-relevant conditions. Specifically, we studied the effect of change in resistivity of a reflector on the backscattered signal of an RDL and investigated an optimal range through simulation. This was followed by the experimental demonstrations of real-time monitoring of Fe film corrosion in pressurized (550 psi) humid CO_2_ conditions. Additionally, remote monitoring of Fe film corrosion in an acidic solution inside a 70 m carbon steel pipe was demonstrated using guided waves. This paper also suggests potential ways to improve the sensing response of IL-RDLs.

## 1. Introduction

Corrosion impacts nearly all industries, including oil and gas (O&G), manufacturing, automotive, and many others that utilize corrosion-susceptible materials [1,2] that create serious safety, environmental, and economic concerns [2]. According to a report published by the National Association of Corrosion Engineers (NACE) in 2016 [3], the global cost of corrosion was estimated to be USD 2.5 trillion per year (3.4% of the global domestic product) in 2013. In another study in 2008, NACE estimated that the total cost of corrosion in the O&G production industry alone is USD 1.372 billion per year [4]. According to their report in 2016 [3], savings of 15–30% of the corrosion costs could be realized if available corrosion control practices were implemented. Efficient monitoring of corrosion-susceptible systems is regarded as an important step in corrosion control and management practices. Accordingly, several techniques such as corrosion coupons, electrical resistance (ER), electrochemical impedance spectroscopy (EIS), and linear polarization resistance (LPR) are available for corrosion monitoring [5]. Despite the availability of these techniques, there exists a gap for continuous real-time and remote monitoring of corrosion in challenging locations.

Sensing techniques based on optical fiber [6,7,8] and surface acoustic wave (SAW) devices [9,10] have the potential to fill this gap. These techniques offer online, continuous, remote, and distributed or quasi-distributed sensing for multiple parameters including metallic corrosion, pH, and gases. While certain types of optical fiber sensors have the capability for fully distributed sensing, their cost and system complexity have been major limiting factors for their widespread use [8]. A network of low-cost wireless and battery-free SAW sensors is an attractive alternative for sensing in remote locations and harsh environments where human access is challenging and electrical connections are impractical [11,12]. These low-cost and highly sensitive devices can provide real-time information about a system being monitored to take action in a timely manner. 

A SAW sensor is a micro-transponder on a piezoelectric substrate fabricated by depositing metallic comb-shaped interdigital electrodes (IDTs) for excitation and detection of SAWs. There are variants of surface wave modes, including Rayleigh wave, shear-horizontal (SH), Love, and many others that have been explored for monitoring temperature, pressure, gases, chemical vapors, pH, corrosion, and others [6,7,13,14,15,16]. For corrosion and pH sensing, SH and Love modes are most suitable for their low attenuation when the sensor is in contact with aqueous media [8,17,18]. In 1990, Arai and Honda [11] showed the monitoring of an aluminum film dissolution process in dilute HCl using a 104 MHz SH-SAW delay line fabricated on 36° Y-X LiTaO_3_. Marquis et al. [19] explored both Rayleigh and SH-SAW devices to monitor the corrosion of copper films in gaseous (air, H_2_S) and liquid mediums. Lately, the use of these devices in combination with an antenna having a sacrificial metal link has been suggested for wireless monitoring of corrosion of metals such as steel in concrete [20,21,22]. Despite these lab-scale demonstrations, their widespread use as corrosion sensors has been limited by several factors, such as high acoustic loss in aqueous media, telemetry challenges, and deployment difficulties.

Here, we investigate impedance-loaded reflective delay line (IL-RDL) SAW devices as potential sensors for continuous and wireless monitoring of corrosion in large systems such as O&G pipelines. 

The proposed sensors were able to wirelessly monitor the corrosion of a 50 nm Fe film under pressurized (550 psi) humid CO_2_ gas conditions in a lab environment as well as in acidic liquid inside a 70 m long carbon steel pipe. Iron was chosen as the sensing element because the intended application is corrosion sensing in carbon steel pipes. Experiments in humid CO_2_ model the corrosive environment in a pipeline conveying CO_2_ for sequestration. Sensors were fabricated on a 36° Y-X LiTaO_3_ substrate with Au metallization and IDTs. A resistive corrosion element of Fe (50 nm) film was connected in parallel with one of the IDT reflectors. Corrosion of the film in humidified CO_2_ under 550 psi pressure and acidic liquid was monitored in real time, wirelessly or in wired mode. The changes in the phase and amplitude of the backscattered signal with the resistance of the proxy film in a corrosive environment were recorded successfully. Long-distance (70 m) wireless interrogation of the sensors inside a carbon steel pipe was achieved using guided electromagnetic waves. 

These initial results validate the concept of wireless corrosion sensing over usefully long distances. Finite element simulations have been performed in order to develop designs for optimized sensors to be studied in future work. In particular, we have determined the optimal value of the corrosion-sensing element. We also propose and validate, through simulations, a self-referencing detection scheme that will simplify interrogation electronics.

## 2. Sensor Design and Simulation

**Figure 1:**
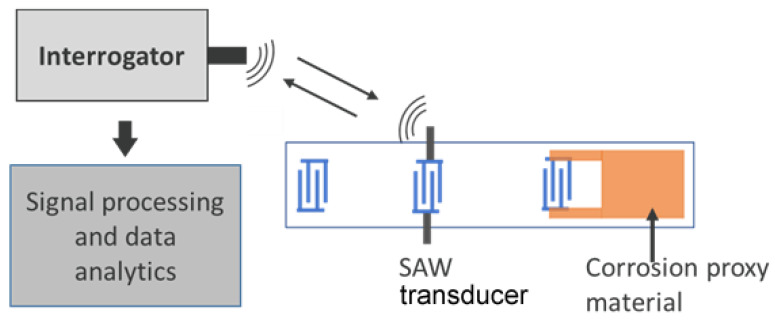
Concept of impedance-loaded SAW corrosion sensor and its interrogation methodology.

Figure 1 shows the proposed sensor design and an interrogation method for continuous monitoring of corrosion of a large system from a remote location. Of specific interest here is an RDL SAW consisting of an emitter IDT at the center and two reflector IDTs on either side. A “corrosion element” in the form of a film, wire, or stripe, made of an application-relevant corrosion proxy material such as iron, carbon steel, stainless steel, copper, etc., is attached to a reflector, as shown in the figure. The propagation path between the emitting IDT and reflectors can be metalized to enhance the propagation of certain modes. When the corrosion element is exposed to a gas or liquid phase corrosive environment, the impedance (resistance or capacitance) of the proxy material changes and induces a change in the amplitude or phase velocity of the backscattered wave at the emitting IDT. This information can be detected by the interrogator as a change in attenuation or phase and used to quantify the corrosion. The impedance of the corrosion sensing element is chosen to have a value that significantly influences the SAW propagation on the piezoelectric substrate. The proposed design has certain advantages:

- Variants of the “sensor element” are possible. The sensor element can be designed as resistive, reactive (inductive, capacitive), or both, depending on the application. The sensing impedance does not need to be located on the piezoelectric substrate, so there is flexibility in using sensor types for other applications in addition to corrosion monitoring.
- Many conventional designs are not suitable for corrosion sensing applications due to the high loss of some modes such as the Rayleigh mode in aqueous media. In this design, SAW propagation paths and metal IDTs can be isolated and/or placed out of the sensing location; any type of SAW mode(such as Rayleigh, SH, Love, etc.) can be used for monitoring corrosion regardless of the sensing medium.
- Corrosion sensing can be integrated with other sensor functionalities (such as gas sensing) in the same transducer regardless of the sensing medium.
- The sensor can be connected directly to the interrogator (wired mode) or can be interrogated wirelessly.

In this study, 36° Y-X LiTaO_3_ and Au were considered as the piezoelectric substrate and metallization and IDT materials, respectively, and Fe film was considered as the corrosion proxy material. Finite element simulations were performed in COMSOL 6.1 or 6.2 to determine the propagation velocities for SAW modes of interest and to predict the changes in the amplitude and phase of backscattered signals as the resistance of the loaded reflector changes. 

**Figure 2:**
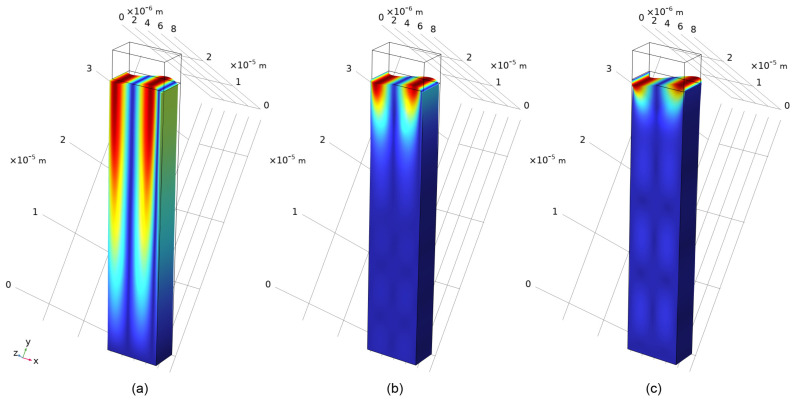
Surface displacements (color) and distorted shape of (**a**) the surface skimming mode, eigenfrequency = 5.2148 × 10^8^ Hz; (**b**) the transverse Rayleigh mode with constant potential boundary condition, eigenfrequency = 5.0943 × 10^8^ Hz; and (**c**) the transverse Rayleigh mode with a 0.15 micrometer gold layer, eigenfrequency = 4.6733 × 10^8^ Hz.

To determine the propagation velocity, a piezoelectric domain of 8 × 10^−6^ m length and 20 × 10^−6^ m height was considered, and eigenmodes were calculated for the domain with periodic boundary conditions. Calculations were performed both with air above the top surface (zero-charge boundary condition) and with a constant potential on the top surface. Figure 2a corresponds to the air termination and yields the surface-skimming mode. Figure 2b has a constant potential on the top and shows a transverse Rayleigh mode. In these figures, the geometry has been rotated with respect to the material coordinate system, and the material coordinate axes are shown. The rotation for 36° Y-X LiTaO_3_ is around the *x*-axis and the eigenmode represents a wave propagating in the *x* direction.

The displacements of these two modes are qualitatively similar; however, the transverse Rayleigh mode decays more rapidly into the substrate. The calculated velocities are 4172 and 4076 m/s. Brzozowski et al. [23] have calculated the wave velocities on free and metalized surfaces and found them to be 4171–4226 m/s and 4077–4112 m/s, with the exact result dependent on the particular source chosen for the material parameters. Our simulated results are therefore consistent with that report. The transverse Rayleigh mode is also obtained when a gold layer of 0.15 × 10^−6^ m thickness is added, although the wave velocity decreases to 3738 m/s (Figure 2c).

**Figure 3:**
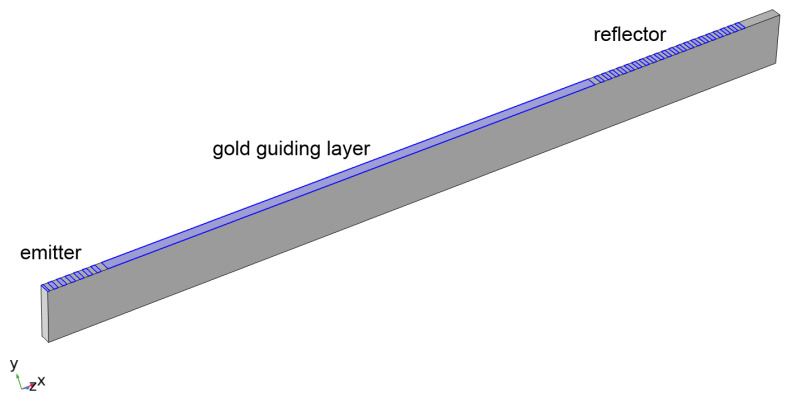
Simulation geometry for frequency-dependent studies.

For the sensor design presented in Figure 1, finite element simulations were performed in the frequency domain in order to determine the amplitude and phase changes resulting from changes in the sensing resistor. The connection between electrodes and the sensing resistor was implemented by coupling the COMSOL electrostatics module to the circuit analysis module. Simulations were performed considering a SAW sensor with an emitter and a reflector with an attached sensing resistor. The simulation domain consisted of half the emitter and one reflector (Figure 3). A symmetry boundary condition was imposed on the left edge and periodic boundary conditions on the front and back surfaces. In order to reduce the size of the problem and the simulation time, the distance between the emitter and reflector was greatly reduced to 248 × 10^−6^ m, and a smaller number of emitter fingers were used (6 driven and 7 grounded electrodes).The reflector consisted of 10 electrode pairs coupled to a sensing resistor. The LiTaO_3_ domain was 5 × 10^−6^ m in depth and 25 × 10^−6^ m high. The right surface and bottom surface were low-reflecting boundaries. The bottom surface was electrically grounded. The guiding layer and IDT electrodes were 0.15 × 10^−6^ m gold (the skin depth of gold is 3.37 μm at 500 MHz, so it is appropriate to consider the entire thickness).

Accurate finite element simulations require a sufficiently small mesh size. For wave propagation problems, a mesh size of λ/5 (where λ is the wavelength of the simulated mode) is considered the minimum adequate mesh size. In the simulations reported here, we have used a mesh size of λ/8 at the sensor operating frequency. We have also performed a mesh size study where we have decreased the mesh size in all dimensions by a factor of 2, increasing the number of mesh elements by a factor of 8. The results obtained were closely similar to those obtained with the larger mesh size. The mesh study, together with the close agreement between the wave propagation velocity and the literature values, supports the simulation approach we have used here.

**Figure 4:**
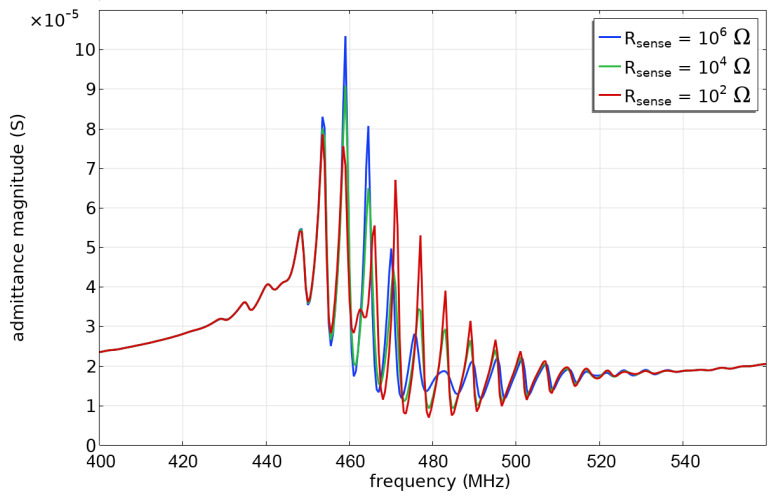

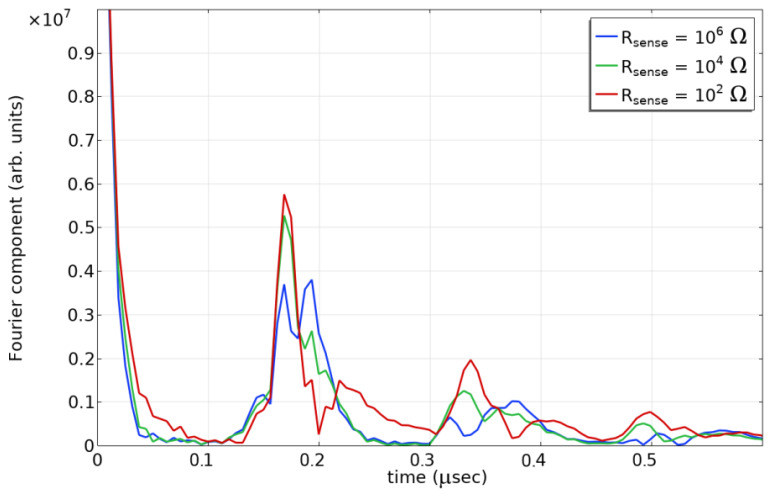
Results from frequency-dependent simulations: (**top**) admittance magnitude at the emitter as a function of frequency for three values of the sensing resistor; and (**bottom**) S11 parameter transformed into the time domain.

We first calculated the reflected wave envelope by simulating the emitter admittance over a range of frequencies around the center frequency (400–560 MHz). From the admittance, the scattering parameter S11 was evaluated and then transformed into the time domain. These simulations were performed for three values of the sensing resistor. Note that the resistor values and simulated admittances are for a domain 5 × 10^−6^ m in thickness and should be scaled for a full-width SAW device. Figure 4(top) is a plot of the admittance magnitude at the emitter as a function of frequency for three values of the sensing resistor. The familiar ripple characteristics of a surface wave reflector are apparent. As the sensing resistance changes, the admittance magnitudes at some frequencies change; some ripples grow and some shrink. From the admittance, we evaluate the magnitude of the S11 scattering parameter which is then transformed into the time domain using the inverse discrete Fourier transform. This is similar to an approach commonly used to analyze experimental data obtained from a vector network analyzer and yields the envelope of reflected pulses. The result is shown in Figure 4(bottom), where the first reflection is apparent at 1.6 × 10^−7^ s, consistent with the round-trip travel distance from the emitter edge to the center of the reflector. Also visible are double- and triple-bounce reflections of smaller magnitude. There is a small change in the peak magnitude and the delay time as the sensing resistor value is changed, with the peak amplitude decreasing and the delay time increasing as the sensing resistance increases.

**Figure 5:**
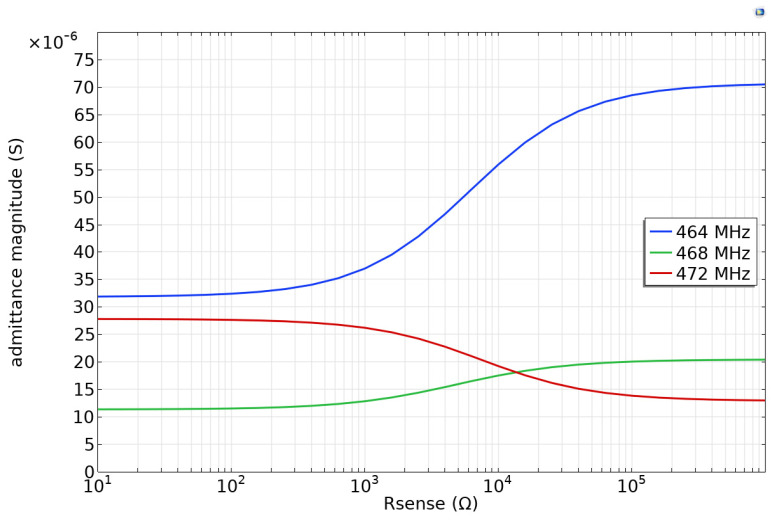
Admittance magnitude of the emitter for three different frequencies as a function of sensing resistance.

Of particular interest is the range of sensing resistor values that causes the full range of delay time and magnitude change. The changes in the envelope in Figure 4(bottom) are a result of the increases and decreases in admittance in Figure 4(top). Consequently, we can use simulations at a single frequency to find the sensing resistor range that causes changes in delay time or reflection magnitude. Figure 5 shows the admittance magnitude for three different frequencies near the emitter center frequency. The magnitude begins to change at about R_sense_ = 100 ohms and is saturated at R_sense_ = 10^5^ ohms. Consequently, for best sensing, one should choose a sensing resistance without corrosion of less than 100 ohms. This value was calculated for an IDT width of 5 × 10^−6^ m, so for an IDT with aperture *W*, the sensing resistor should be less than (5 × 10^−6^ m/W[m])×100 ohms. In the experimental results presented later, the SAW device had an aperture of 1.6 mm; consequently, an uncorroded resistance of 0.31 ohms is optimal. 

**Figure 6:**
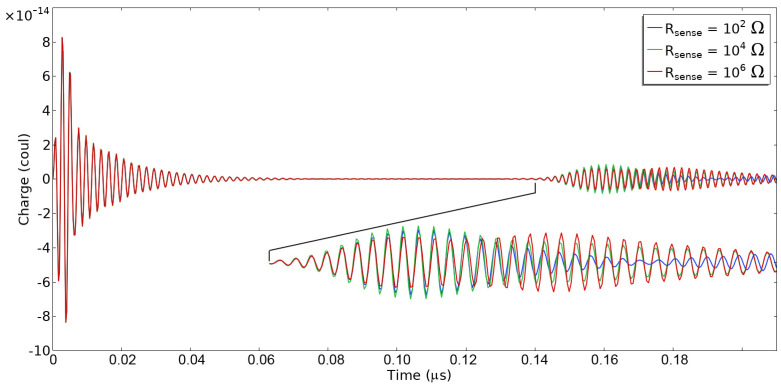
Transient response to a sinusoidal pulse centered at 468 MHz for three different values of the sensing resistor.

To achieve better insight into the phase and time delay changes due to corrosion, we performed transient simulations. The simulations were performed in the time domain with the same conditions used for the frequency-dependent simulations except for a domain length of 226 × 10^−6^ m. The emitting IDT was excited by a 3-cycle windowed sinusoid with a center frequency *f*_0_ = 468 MHz. The time step was 1/8·*f*_0_, yielding a sufficient number of steps per sinusoidal period, and “strict” time-stepping was used. Figure 6 shows the simulation results for three resistance values. The emitter is resonant with an underdamped quality factor, so the initial ringing takes some time to decay, although in this case, the ringing does not prevent observation of the reflected pulse. The reflected pulse reaches a peak at a slightly shorter time for a small value of the sensing resistance, consistent with the frequency domain simulations. There is a consistent phase shift between shorted and open terminations of about 70 degrees. The predicted changes in the magnitude and phase of the reflected waves associated with the varying sensing resistance are small for 36° Y-X LiTaO_3_ devices. These results are consistent with the data reported in the literature. The literature [24,25] reports a small change in the reflection amplitude (a few dB) and phase (~45 degrees) associated with the variable resistive loading. Liu et al. [16] have used the P-matrix approach to predict the effect of loading on the SAW reflection or a YZ-LiNbO_3_ sensor. Luo et al. [17,26] have performed measurements on a loaded 128° Y-X LiNbO_3_ sensor. Tsai et al. [27] have performed an analysis using the coupling of modes model.

## 3. Experimental Results

For experimental demonstrations, RDL devices of 16 µm wavelength and 1.6 mm aperture consisting of an emitter IDT (30 finger pairs) and two reflectors (10 finger pairs) were designed such that the emitter was at the middle and the reflectors were displaced by 2.516 mm and 3.915 mm (Figure 1). The devices were fabricated on 36° Y-X LiTaO_3_ by patterning 150 nm thick Au IDTs and metallization layers using standard photolithography and lift-off processes. The operating frequency of the fabricated devices was 256 MHz. An iron film of thickness 50 nm, width ~0.5 mm, and length 2 mm was sputtered after fabrication of the devices. The estimated value of the resistance of this iron film is 7.14 ohms (1/σ) × (2 mm)/(0.15 × 10^−6^ m) × (0.5 mm), where σ represents the conductivity. The measured S11 characteristics of a representative device in the time domain had peaks at 1.32 µs and 2.12 µs (not shown here), near to the expected values based on the propagation velocity of the SH-SAW mode. 

**Figure 7:**
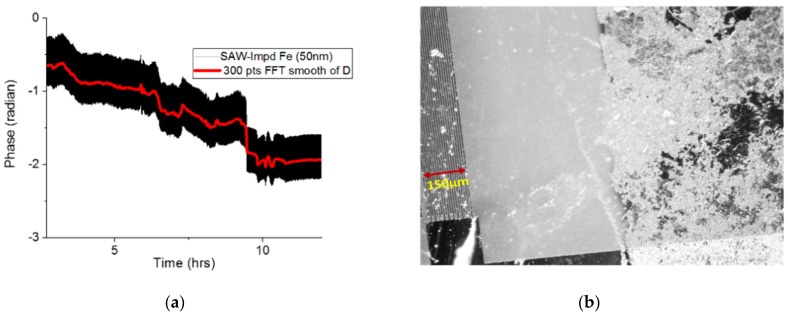
Experimental results from an Fe 50 nm coated IL-RDL sensor: (**a**) detected phase as a function of time, and (**b**) optical image of the film after the corrosion experiment.

To monitor the corrosion of the Fe film under pressurized humid CO_2_, a device was mounted on a glass surface using epoxy, and electric contacts of copper wires were made using a conducting paste followed by an epoxy layer. The epoxy layer was used to improve the mechanical stability as well as protect the contacts in the corrosive environment. The devices were then installed in a tube reactor capable of holding 1000 psi pressure using a pass-through that was isolated from the high-pressure fitting and the reactor’s wall. The devices were interrogated using a custom-assembled interrogator (NI-PXI), and real-time data were collected in wired mode or wirelessly [28]. Wireless interrogation was carried out using a pair of half-wavelength 256 MHz dipole antennas. The sensors were exposed to dry N_2_ (30 min) and dry CO_2_ (30 min) to create a baseline before being exposed to the humid CO_2_ overnight. All experiments were performed at 550 psi pressure. Figure 7a shows the detected phase of the reflection corresponding to the IDT connected to the iron strip as a function of time. The sensor phase began decreasing at around 4 h of exposure to the wet gas and decreased monotonically, indicating the progress of the film’s corrosion. The impedance of the sensing load can be resistive or capacitive [16,29], affecting the amplitude and phase change differently. Here, resistance change dominates the capacitance change as the corrosion progresses. Figure 7b shows an optical image of the sensor after 24 h of exposure to the corrosive environment that clearly shows the corrosion of the film. In a previous study [30], we reported that an Fe film of similar thickness coated on an optical fiber under pressurized CO_2_ conditions had a corrosion onset in about four hours. Chemical analysis of Fe film corrosion in humid CO_2_ was given in that study [22].

**Figure 8:**
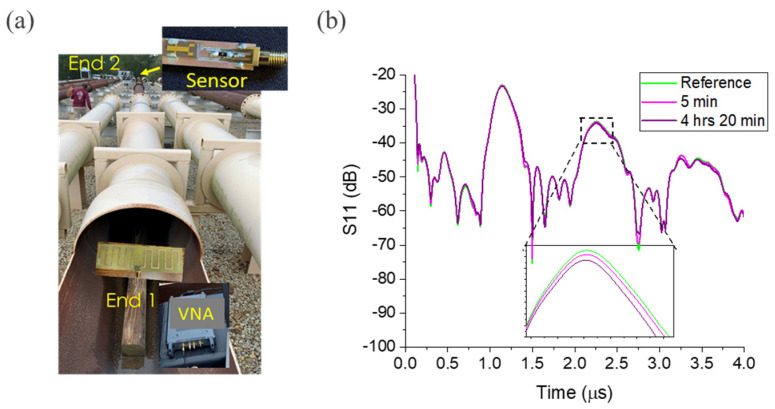
Corrosion sensor testing: (**a**) 70 m long carbon steel pipe with both ends open used for this experiment; and (**b**) scattering parameter S11 transformed into the time domain of an Fe (50 nm)-loaded sensor when immersed in acidic water. The sensor was mounted at “End 2” of the pipe and wirelessly interrogated from “End 1” using TE11 mode of guided waves inside the pipe.

In addition to the lab test, the capability of the sensors for remote monitoring of in-pipe corrosion was tested in a 70 m long, 30″ outer diameter carbon steel pipe with a 0.25″ thick wall and open ends (Figure 8a). The pipe had multiple flanged sections bolted together, and the ends were open to the environment. It was located at Lazy Q Ranch, La Grange, TX, a test facility of Quanta Services. The sensor was attached to a 256 MHz dipole antenna and placed at one end of the pipe and was interrogated from the second end using a vector network analyzer (VNA) via another 256 MHz dipole antenna. The exciting power was 5 dBm. The wireless interrogation inside the pipe was performed in the guided TE11 electromagnetic wave mode [31]. Figure 8b shows the backscattered signal of the sensor recorded at different times after a corrosive (acidic) solution was dropped on the iron film. As shown in the inset, the attenuation and time delay (or phase) of the backscattered signal changed with time under the acidic solution and the change became prominent in about 4 h. No film was observed after the test under visual inspection. The measurements in the pipe showed a longer delay time for the IDT reflection consistent with the pipe length and the TE11 wave velocity.

The changes in reflection magnitude and phase are consistent with our simulations and previous publications [16,17]. The magnitude change is small and the phase change, although significant, can be challenging to detect in the presence of other environmental factors. Accordingly, we suggest in the following section a method for simplifying the detection process.

## 4. Self-Referencing Detection 

We propose here a novel sensor design that greatly simplifies the detection of corrosion. We noted above that there was only a small change in the magnitude of the reflection and a phase change of order 70 degrees. Resolving this phase change requires a reference reflection, which is usually obtained from a second IDT with no corrosion element. This second IDT must be placed at a different distance so that its reflection can be distinguished from the IDT loaded by the corrosion element. However, there are also phase shifts resulting from temperature or other environmental changes, adding additional complications. 

**Figure 9:**
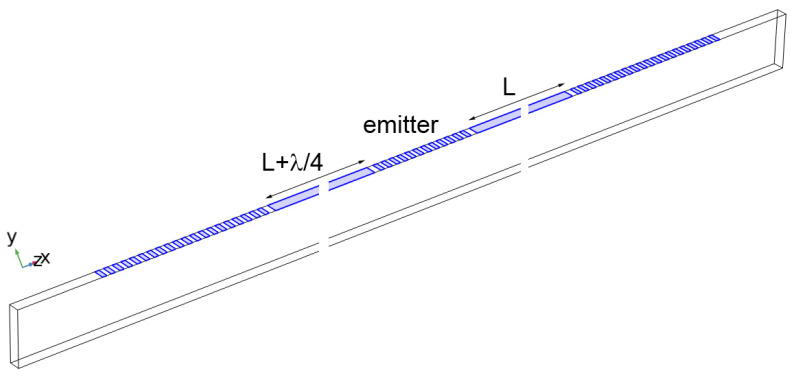
Self-referencing corrosion sensor.

Consider instead an emitting IDT with two reflecting IDTs located on opposite sides (Figure 9). One of these IDTs is displaced an additional quarter-wavelength away from the emitter. Suppose one of these detectors is shorted and the other is connected to the corrosion element. 

When the element is uncorroded, we have two identically loaded IDTs with one having a path length for the reflected pulse greater by a half wavelength. The two reflections will be added at the emitter, as one of the reflections first encounters a grounded electrode and the other a driven electrode. Corrosion of the sensing resistor contributes an additional phase shift to one reflection and a nonzero signal will be observed at the emitter. 

**Figure 10:**
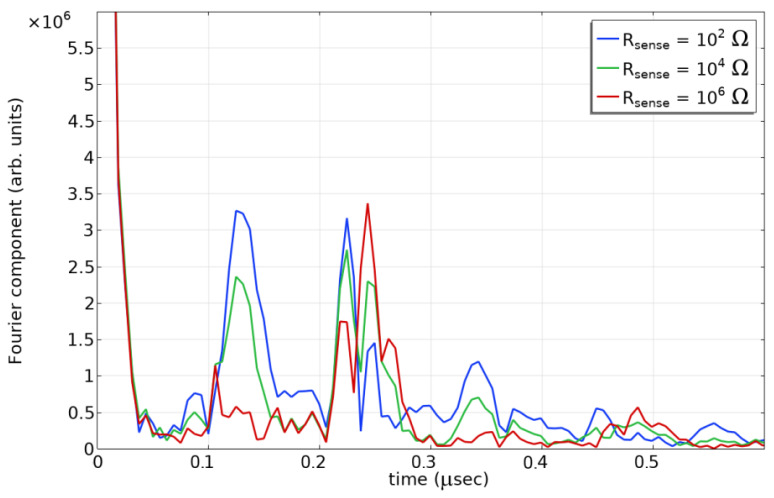
Simulated reflection envelope for three values of the sensing resistor.

We illustrate this detection scheme with simulations in Figure 10. The emitting IDT has six pairs and the reflecting IDT has ten pairs of electrodes. The closer IDT (*L* = 154 × 10^−6^ m) is shorted and the second (*L* + λ/4 = 156 × 10^−6^ m, where λ represents wavelength) is terminated in a variable resistance representing the corrosion element. As before, the admittance was simulated over a range of frequencies, used to calculate the S11 parameter, and transformed into the time domain. 

The first reflection at about 1.2 × 10^−7^ s is strong when the sensing resistor is uncorroded (R_sense_ = 100 Ω) and disappears when it is fully corroded. As a result, it will be easy to distinguish between uncorroded and fully corroded by a threshold for the magnitude of this reflection. Note that one concern might be a failure of the sensor or interrogation apparatus. Correct operation can be verified by the existence of a double-bounce reflection at about 2.3 × 10^−7^ s. This reflection will always be nonzero because some double-bounce paths incur an additional path length of a full wavelength λ.

## 5. Summary

We investigated the potential of using impedance-loaded surface acoustic wave devices as corrosion sensors through simulations and initial experimental studies. The simulation studies showed that the magnitude and time delay of the sensor devices change with a resistive load, and there exists a range of the resistive load for optimal performance of the sensor. We demonstrated the ability of the sensors to wirelessly monitor metal corrosion in an aggressive environment, such as humid CO_2_ gas at high pressure and acidic solutions inside long pipes. Finally, a self-referencing sensor design has been proposed and simulated, which simplifies the detection signal processing. These observations pave a path to developing remote sensors for real-time monitoring of internal corrosion in large infrastructures consisting of metallic pipes.Future work will use the results from finite element simulations to determine the optimal value of the corrosion sensing resistance. We also hope to explore the impact of different thicknesses on the sensing element. The proposed self-referencing detection scheme is expected to considerably simplify the interrogation electronics.

As this type of corrosion sensor can be interrogated over long distances, it is appropriate to consider the possibility of interrogating multiple sensors from the same location. This should be possible for a small number of sensors by using frequency or time diversity; that is, sensors that differ in center frequency or propagation path length. Interrogation of multiple sensors is another issue that may well be investigated in future work. 

## Data Availability

Some simulation and data files can be obtained by contacting the authors.
